# Classifying Sources Influencing Indoor Air Quality (IAQ) Using Artificial Neural Network (ANN)

**DOI:** 10.3390/s150511665

**Published:** 2015-05-20

**Authors:** Shaharil Mad Saad, Allan Melvin Andrew, Ali Yeon Md Shakaff, Abdul Rahman Mohd Saad, Azman Muhamad Yusof @ Kamarudin, Ammar Zakaria

**Affiliations:** 1 Center of Excellence for Advanced Sensor Technology (CEASTech), Universiti Malaysia Perlis (UniMAP), Taman Muhibbah, Jejawi, 02600 Arau, Perlis, Malaysia; E-Mails: allanmelvin.andrew@gmail.com (A.M.A.); aliyeon@unimap.edu.my (A.Y.M.S.); ammarzakaria@unimap.edu.my (A.Z.); 2 Faculty of Engineering Technology, Universiti Malaysia Perlis (UniMAP), Kampus UniCITI Alam, 02100 Sungai Chuchuh, Padang Besar, Perlis, Malaysia; E-Mails: abd.rahman@unimap.edu.my (A.R.M.S.); azman@unimap.edu.my (A.M.Y.K.)

**Keywords:** indoor air quality, artificial neural network (ANN), pattern recognition

## Abstract

Monitoring indoor air quality (IAQ) is deemed important nowadays. A sophisticated IAQ monitoring system which could classify the source influencing the IAQ is definitely going to be very helpful to the users. Therefore, in this paper, an IAQ monitoring system has been proposed with a newly added feature which enables the system to identify the sources influencing the level of IAQ. In order to achieve this, the data collected has been trained with artificial neural network or ANN—a proven method for pattern recognition. Basically, the proposed system consists of sensor module cloud (SMC), base station and service-oriented client. The SMC contain collections of sensor modules that measure the air quality data and transmit the captured data to base station through wireless network. The IAQ monitoring system is also equipped with IAQ Index and thermal comfort index which could tell the users about the room’s conditions. The results showed that the system is able to measure the level of air quality and successfully classify the sources influencing IAQ in various environments like ambient air, chemical presence, fragrance presence, foods and beverages and human activity.

## 1. Introduction

People normally spend most of their time in indoor environments. Therefore, their health depends heavily on the indoor environment in which they live. Hence, meticulous attention should be given to make sure the indoor environment is safe and comfortable. As a major part of the indoor environment, attention should also be given to indoor air quality (IAQ). Continuous monitoring of IAQ is important to make sure people breathe in a healthy and safe air. Real-time IAQ monitoring keeps people alert to any pollution that might be present in an indoor environment right as it happens. A good IAQ monitoring system should also be able to tell the users about the source of pollutants (for example: volatile organic compounds (VOCs) emitting from a chemical product). A better IAQ monitoring system with enhanced featured is proposed in this paper—a smart IAQ monitoring system. This smart IAQ monitoring system could identify and inform the users about the source influencing the IAQ level. For example, when there is smoke in a room, this system could identify the instance and inform the users. The information is sent through wireless network to a database where the users can access it from anywhere.

In order to make sure that the level of IAQ is within the acceptable level, many parties especially building administrators have made a considerable effort. Some researchers found that low level of IAQ could affect the quality of life of the occupants and may result in low productivity [1]. Modern buildings, especially, need more attention on IAQ because these buildings have been built with the concern of energy conservation [2]. Following the oil crisis in the late 70s, the American Society of Heating, Refrigerating, and Air-Conditioning Engineers (ASHRAE) had come out with certain guidelines for new buildings designed to be energy efficient [3]. This change in the practice of modern buildings however has increased the possibility for building occupants to develop Sick Building Syndrome (SBS) and some other Building Related Illness (BRI).

The standards for IAQ have been issued by various parties such as ASHRAE, WHO, US EPA and by individual country like Malaysia, Singapore and Hong Kong. For the purpose of this study, the IAQ standards issued by US EPA and ASHRAE have been followed closely. IAQ, as defined by the ASHRAE in Standard 62-2001: Ventilation for Acceptable Indoor Air Quality, is air in which there are no known contaminants, as harmful concentrations are determined by cognizant authorities and with which a substantial majority (80% or more) of the people exposed to it do not express dissatisfaction. Even though IAQ problems happen occasionally, it could be severe when they do happen. The studies conducted by the United States Environment Protection Agency (US EPA) showed that pollutants present in indoor environments sometimes can be more dangerous than pollutants in outdoor environments. In most cases, the concentration of pollution was usually about five times higher than the concentration level found outside. However, in severe cases, the concentration of pollution could be 100 times higher [1]. Among the most common indoor air pollutants include gases, chemicals and living organisms like mold and pest. These pollutants could impair the health of the building occupants. Sore eyes, headaches and fatigue are among the common complaints associated with low IAQ level. In serious cases, occupants always complain about respiratory related illness, heart disease, and some may even suffer from cancer and other serious health problems [4,5]. At a very high concentration, pollutants like carbon monoxide can lead to death [6].

This study aims to propose a smart IAQ monitoring system where the sources influencing IAQ can be identified. This study uses ANN to train the system to recognize the sources influencing the IAQ level in five conditions: ambient air, chemical presence, fragrance presence, foods and beverages presence and human activity. In artificial intelligence (AI) field, ANN is one of the major components which use a modeling technique inspired by the human brain (memory-less processing elements known as neurons or nodes which are nonlinearly interconnected) and which can be trained from examples. When ANN is properly trained, it can cleverly classify a data to a population. This attribute of ANN is so special that it may give better results as compared to other techniques in the same situation. Therefore, many researchers have opted for the use of ANN including in environmental sciences—usually to predict atmospheric concentration of NO_2_, Ozone, benzene and PM_10_ [7,8,9].

This IAQ monitoring system consists of three parts: sensor module cloud, base station and service-oriented client. The sensor module cloud (SMC) contains collections of sensor modules that measure the air quality data and transmit the captured data to base station through wireless network. Each sensor modules includes an integrated sensor array that can measure indoor air parameters like Nitrogen Dioxide (NO_2_), Carbon Dioxide (CO_2_), Ozone (O_3_), Carbon Monoxide (CO), Oxygen (O_2_), Volatile Organic Compounds (VOCs) and Particulate Matter (PM_10_) along with temperature and humidity. 

## 2. Related Works

Some researchers have proposed a framework or a system to monitor IAQ using wireless sensor networks such as [10,11,12], while others have incorporated index calculation to determine the IAQ level such as [13,14,15].

Hui Xie *et al.* [7] exploited the ANN in order to predict the SBS. The results were compared to the other linear regression models. It was concluded that ANN gave better predictions and could be useful in other areas as well. Ghazali *et al.* [16] also developed ANN air quality prediction model using feed-forward network. The result is similar to Hui Xie *et al.* [7] where the model with ANN structure produced the best prediction. Abd Rahman *et al.* [17] proposed a forecasting of air pollution index. The forecasting was based on 10-year monthly data of Air Pollution Index (API) located in industrial and residential monitoring stations area in Malaysia. The autoregressive integrated moving average (ARIMA), fuzzy time-series (FTS) and ANN were used to forecast the API values. The results showed that ANNs gave the smallest forecasting error as compared to the other two methods. Clearly, ANN is useful in decision making processes for air quality control and management.

Previous researches on IAQ monitoring system usually used the ANN to predict or forecast either the value of air quality or the value of air pollution. This project is different from previous researches because this project aimed at training the ANN to classify and identify the sources that influence the indoor environment. The functionality of this system and the methodology of this project are explained in the next section.

At present, odour has also been included as part of sources influencing IAQ [18]. Therefore, many studies had been carried out to use the IAQ monitoring system for odour detection in real time. In fact, the IAQ monitoring system is the only system available to get continuous, quick and reliable information about the presence of odours in ambient air [18]. However, the odour recognition principle was mainly used to investigate the odour in outdoor environments [19,20,21,22]. In indoor environments, odour recognition was usually used to detect odour from one single category. For example, the use of odour recognition to recognize types of mushrooms, oil flowers and pure chemicals only [8,15,23]. Nonetheless, odour recognition from sources of indoor air pollutants has not been carried out.

## 3. Selection of IAQ Parameters

There are various parameters involved in measuring IAQ. These parameters are divided into four categories: physical condition, chemical contaminants, biological contaminants, and other common contaminants. However, for the purpose of this project, only nine parameters have been chosen. These parameters have been identified as the parameters that are most used in measuring the IAQ [24,25,26,27]. The parameters that are used in this project include parameters of common indoor air contaminants (CO_2_, CO, O_3_, NO_2_, VOCs, O_2_ and PM_10_) and thermal comfort parameters (temperature and humidity).

## 4. System Architecture

**Figure 1:**
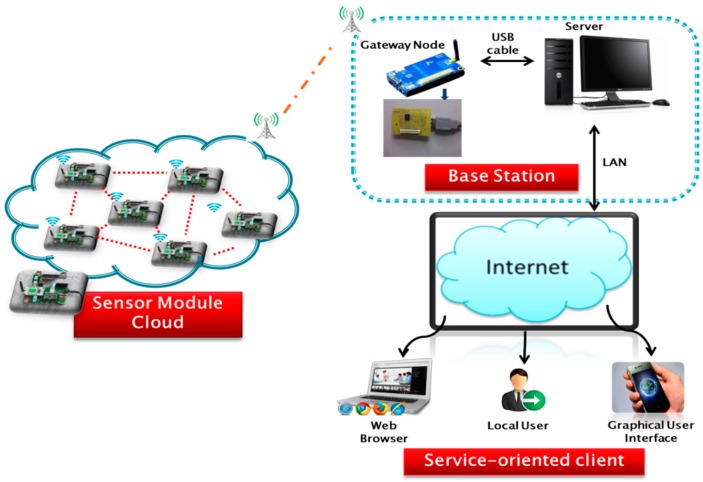
System architecture for real-time IAQ monitoring.

The proposed system has been designed in such a way that it is able to monitor air quality in both indoor and outdoor environments. Nine sensors have been used to capture data for the nine parameters required in this project. Figure 1 shows our proposed system architecture for real time IAQ monitoring. The proposed system consists of sensor module cloud (SMC), base station and service-oriented client. The SMC contains an array of sensor modules that captures the air quality data. The data are then transmitted to the base station through wireless connection where the data are stored in a server. The server functions as data logger to keep track of data received in base station. It stores the data in database, processes the data, performs analysis and provides information about IAQ information through web service. The web service enables the clients or users to be informed about the IAQ level in real-time.

### 4.1. Sensor Module

**Figure 2:**
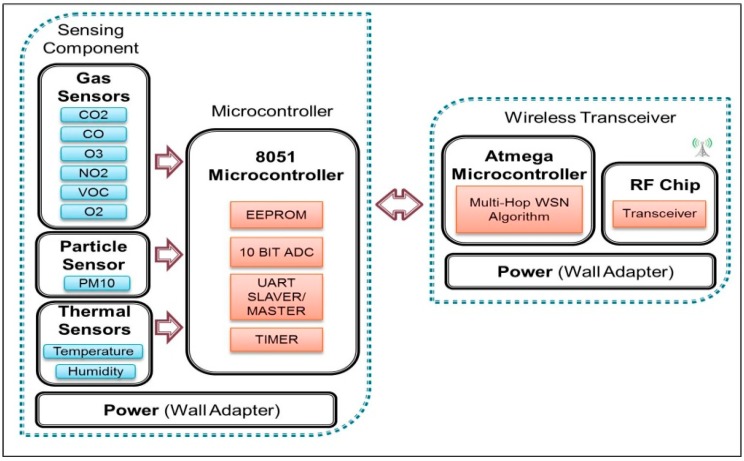
Block diagram of the sensing node.

The sensor module is used to sense and measure air quality in indoor environment. Figure 2 depicts a schematic diagram of the major components of the sensing module. Basically, there are three major components: sensor components, microcontroller and wireless transceiver. This entire component is powered-up by using wall adaptor for continuous power supply. 

In this study, a microcontroller unit (MCU) named STC12C5A32S2 with built in 10 Bits Analog-To-Digital Converter (ADC) converts the analogue reading for each sensor response to digital value. STC12C5A32S2 is chosen because this 8-bit microcontroller processes 12 times faster than general 80C51 (it can go up to 35 MHz operation frequency) [28]. After the conversion of the sampling voltage by ADC, the microcontroller then is responsible for encapsulating the converted data into packets and passing the packets via serial port to the wireless transceiver.

**Figure 3:**
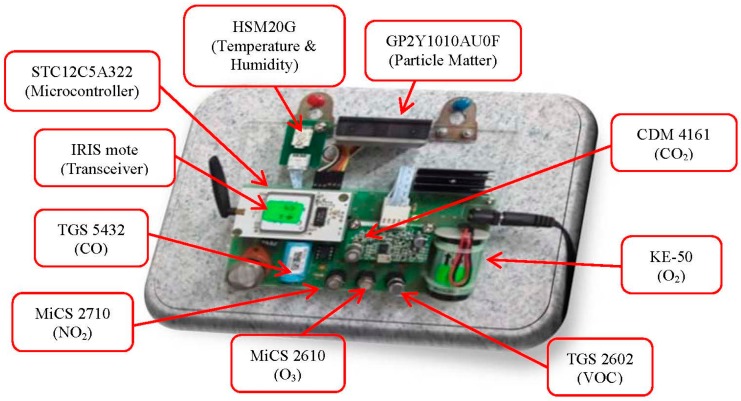
Prototype of sensor module.

The wireless transceiver unit transmits the data packets by using Multi-Hop Wireless Sensor Network (WSN) algorithm through the Radio Frequency (RF) chip. Implementation of WSNs in IAQ monitoring has reduced the installation costs due to wire depositions. The IRIS mote from Memsic Company has been selected as the wireless transceiver unit for its low power consumption, low price and complies to the IEEE 802.15.4 wireless protocol [29]. The operating system used in this mote is based on TinyOS which allows the user to quickly implement the communication network [30]. In this system, each IRIS mote is responsible to receive data from sensor components through microcontroller. The data from IRIS is sent to the base station. However, if there is another IRIS mote nearer to the base station, the data is sent to its neighbour (the other IRIS mote) before sending the data to the base station. This data transmission process is known as multi-hopping process. Figure 3 shows the prototype sensor module, consisting of nine gas sensors, and wireless transceiver with microcontroller attached to the wireless transceiver.

The sensor components consist of three types of sensors: gas sensor, particle sensor and thermal sensor. Lists of sensors used in the proposed system along with their operational range are presented in Table 1 below. Each sensor generates voltage signal based on current environment. These sampling voltage levels are read by microcontroller periodically.

**Table 1:** List of sensors used in the system.

No	Sensor Name	Sensor Type	Manufacturer	Target Gas	Typical Detection Range
1	CDM 4161	MOS	Figaro	CO_2_	400–2000 ppm
2	TGS 5342	Electrochemical	Figaro	CO	0–100 ppm
3	TGS 2602	MOS	Figaro	VOCs	0–30 ppm
4	MiCS-2610	MOS	SGX Sensortech Limited	O_3_	0.01–1 ppm
5	MiCS-2710	MOS	SGX Sensortech Limited	NO_2_	0.01–5 ppm
6	KE-25	Electrochemical	Figaro	O_2_	0%–100%
7	HSM20G	Thermal	GeeTech	Humidity	20%–95% RH
		Thermal		Temperature	0–50 °C
8	GP2Y1010AU0F	Optical	SHARP	PM_10_	0–0.5 mg/m^3^

Selecting a proper gas sensor is a relatively complicated issue as many factors need to be taken into consideration. For this study, most of the gas sensors are metal oxide based, while the rest are electrochemical based. These Metal Oxide Semiconductor (MOS) sensors contain tin dioxide (SnO_2_) sensing element that responds to the gas molecules, which are typically volatile compounds [31]. It consists of two major parts, namely the heater and sensor substrate. The substrate has two terminals and its resistance is measured as a representation of the amount of gas concentration in the environment while the heater provides the stabilized temperature needed for the measurement [32]. Due to its long lifetime, high sensitivity response and low cost, this type of sensor is commonly used in many indoor applications such as homes, offices and factories appliances. The second type of gas sensor used in this study is electrochemical based sensor. This type of sensor has high sensitivity to environmental change and it does not need power to operate. 

However, this type of sensor has its limitations. These low cost sensors cannot provide accurate readings of the gas presence in the air since they are strongly affected by the temperature, humidity and the presence of other gases. To compensate this limitation, the patterns are observed instead of the actual data measured. The sensor resistance and the gas concentration in the environment interact in the following expression.

(1) Rs= A(C)− α

where *Rs* is the sensor resistance of the sensor, *A* is constant, *C* is the gas concentration and α is the slope of *Rs* curve [33].

To detect the physical contaminant like dust or particulate matter, Sharp GP2Y1010 optical dust sensor from Sharp has been chosen. This sensor contains an infrared emitting diode and a phototransistor which are arranged diagonally. Any dust that gets into the sensor makes the infrared light reflect which can be detected by phototransistor. The advantage of this dust sensor is it can detect small particles like cigarette smoke. It is commonly used in an air purifier system because it is small, cheap, robust and uses low current consumption [34]. For thermal sensor, HSM-20G sensor is used to detect temperature and humidity. HSM-20G is an analog sensor that converts the ambient temperature and relative humidity into standard output voltages (1–3.3 V). It can measure the temperature between the ranges of 0–50 °C. It is also able to detect the relative humidity (RH) from 20% to 95% with accuracy ±0.5% at 25 °C [35]. With the calibration curve provided in the datasheet, these analog voltages can be converted to the unit of temperature (°C) and relative humidity (%).

### 4.2. Base Station

**Figure 4:**
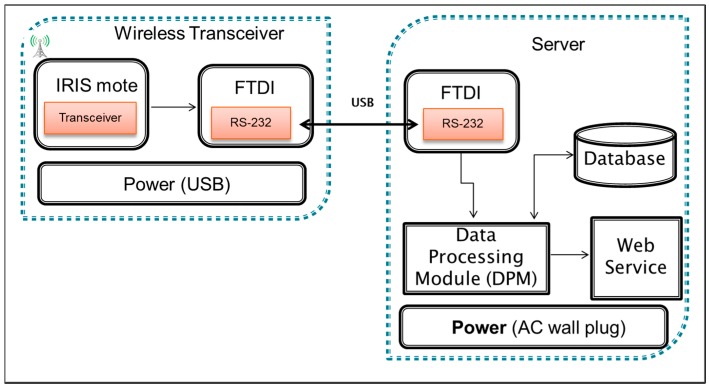
Block diagram of base station.

The base station in IAQ monitoring system contains two components which are wireless transceiver and a server that act as a data logger. The base station is responsible for managing, collecting and recording the data before displaying it on a computer screen and on the web service. The wireless transceiver unit is similar to the sensor module, which contains IRIS mote—ATmega1281 low power microcontroller and AT86RF230 radio frequency (RF) [29]. It also contains Future Technology Devices International (FTDI) device which emulates RS 232 transmission protocol and communicates with Data Processing Module (DPM). DPM is responsible for processing and writing the air quality data into the database. At the same time, DPM sends the data to the Web Service which allows users to access the information in real-time. A simple database is based on a SQLite format which is used to log the data for further processing (if necessary) on the server system. SQLite has been selected instead of alternatives like MySQL simply due to the fact that it is easier to setup and it uses single file storage. However, due to its limitation of storage, the DPM has been programmed to create one SQLite file which contains one week of data, and this process is repeated for the following weeks. Figure 4 shows the block diagram of the base station.

### 4.3. Service-Oriented Client

**Figure 5:**
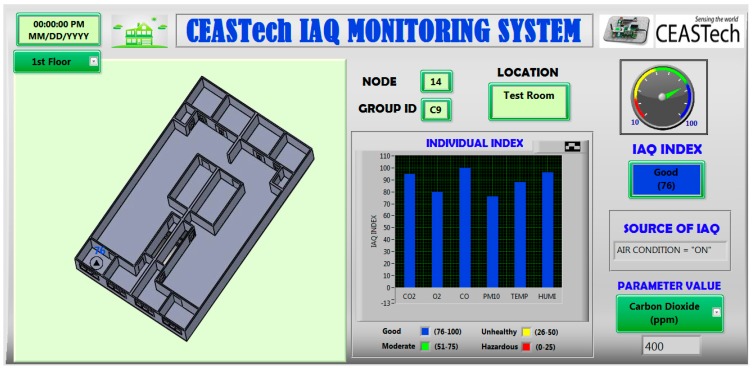
GUI visualization for IAQ monitoring system.

Service-oriented client provides all information on IAQ accessible in real-time. The data is shared through self-developed Graphical User Interface (GUI). The GUI is the interface that facilitates the users to interact with the programs. In this research, the GUI was developed by using LABVIEW software program. In order to stream sensor data for web service that was located in the server, the GUI adopted websocket technology. Figure 5 shows the visual of GUI that has been developed.

This GUI provides a map location of the sensor modules which are placed at different locations such as meeting room, lecture room and postgraduate room (this study uses CEASTech Institute in UNIMAP, Perlis as its base location). It also provides the current value IAQ parameters with color-coded graph based on IAQ index level. The system receives the data in the forms of voltages. These voltages are compared to the specified ANN output model that has been trained and then the data are classified according to the training set (any one of the five categories trained). The GUI then displays the information of the source of activity influencing IAQ level.

## 5. Development of IAQ Index (IAQI)

Indoor environmental index has been developed to identify the quality of indoor air as well as the comfort level of the occupants inside a building. To achieve this, two separate indices have been developed: indoor air quality index (IAQI) and thermal comfort index (TCI). Both indices are divided into four status categories as shown in Table 2 below. The indoor environmental index was actually modified from the U.S. EPA Air Quality Index (AQI) which was used for outdoor air so that it fits with the indoor air parameters. 

**Table 2:** Index values and status categories.

Index Value	Status	
	IAQI	TCI
76–100	GOOD	MOST COMFORT
51–75	MODERATE	COMFORT
26–50	UNHEALTHY	NOT COMFORT
0–25	HAZARDOUS	LEAST COMFORT

## 6. Methodology

### 6.1. Calibration and Validation

**Figure 6:**
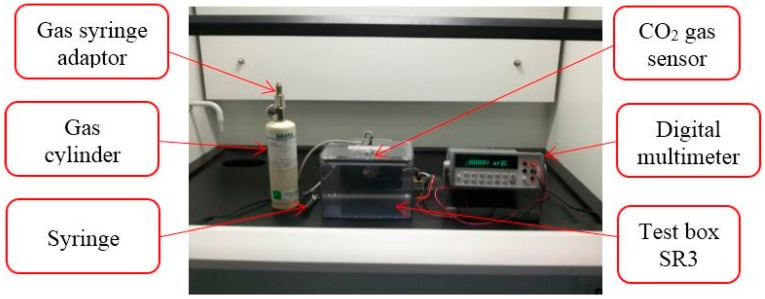
Calibration setup.

Calibration of gas sensors is one of the main challenges during the development phase. Although calibration was carried out for all sensors, the discussion was limited only to the calibration of CO_2_. Calibration of the CO_2_ sensor was performed in a laboratory environment with each sensor mounting in a completely sealed gas test box SR3 (manufactured by Figaro) specially designed for the testing of gas sensors to avoid any other gases affecting the experiment. The size of the box is 235 mm × 180 mm × 210 mm. Figure 6 shows calibration setup for CO_2_ gas sensor which was set in the test box.

**Figure 7:**
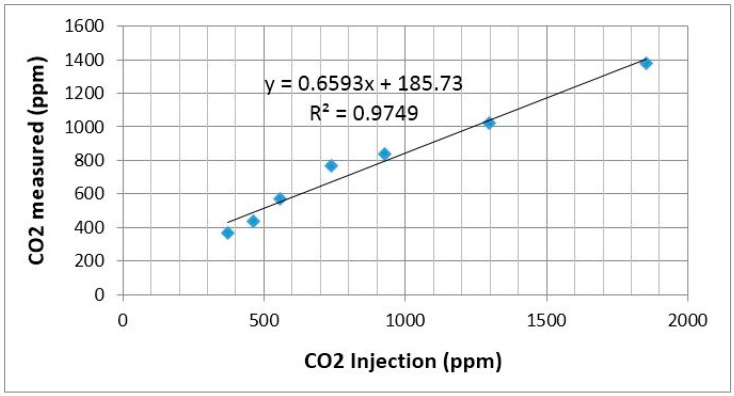
Scatterplot of CO_2_ sensor calibration.

For the use of the SR3 box, the method specified by the manufacturer was used. Initially, the test box was left open in a clean environment and mixing fan was turned on for 3 min to ensure that all contaminants had been removed. After that, a lid was put on the box. Subsequently, the syringe was filled with a volume of CO_2_ extracted from a gas syringe adaptor of the gas cylinder. High purity gas (≥99.995%) was used for the calibration. CO_2_ gas was injected in the box through a silicon septum and the mixing fan was turned on for 30 s. A time lapse of 30 s was allowed before reading the sensor output. The lid of the box was removed so that it could return to the 3 min cleaning cycle. During the experiment, room temperature of the calibration environment was maintained at 25 °C. Figure 7 shows the scatterplot of CO_2_ sensor calibration result at different CO_2_ gas concentrations.

It was observed that the output of the gas sensor was linear to the gas sample concentration. Simple linear regression (LR) method was used as the calibration method for the gas sensors. The coefficient of determination (*R*^2^) was 0.97 indicating that the value was a good correlation between the measured data to the gas injected. This data was used to recalibrate the equations provided by the manufacturer in the datasheet.

**Figure 8:**
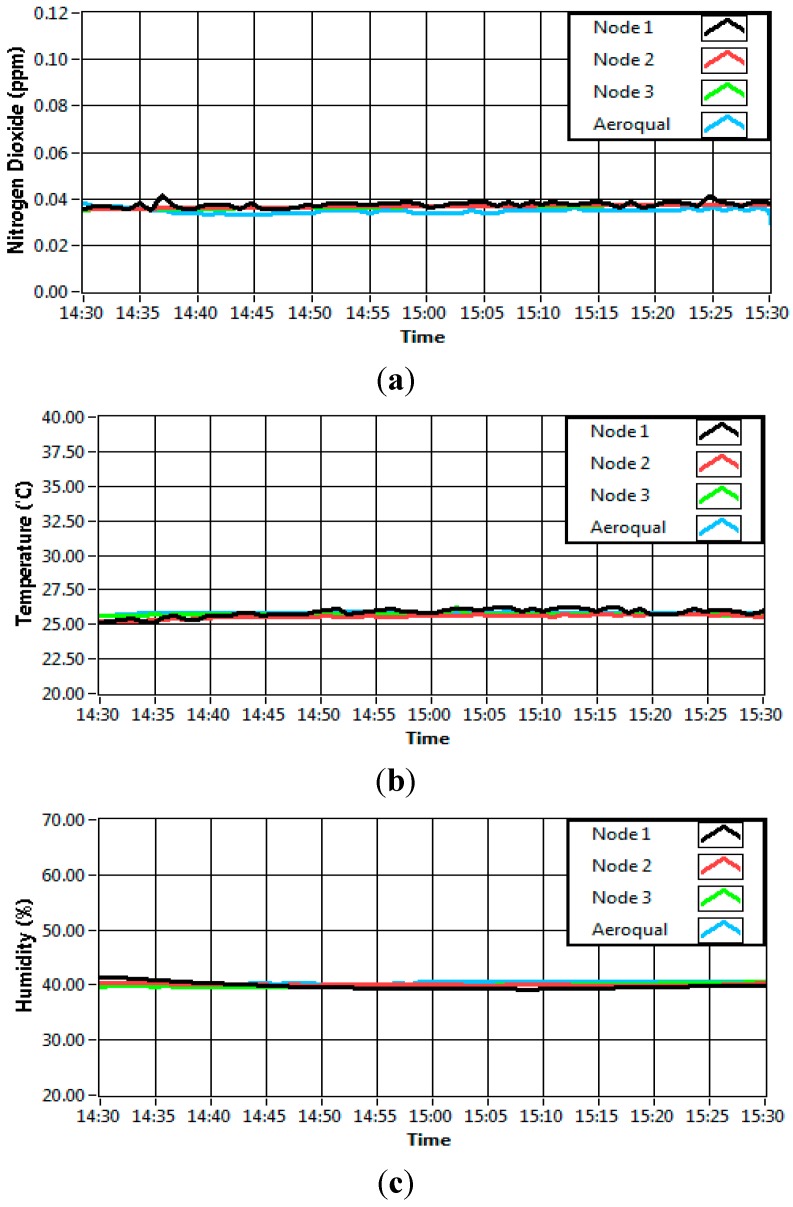
(**a**) NO_2_ data; (**b**) Temperature data; (**c**) Humidity data.

Validation procedure had also been carried out to make sure that the data collected by the self-developed sensor node was similar to the data collected by a commercialized device. The discussion of this procedure was limited only to the NO_2_, temperature and humidity sensors. The sensor validation was carried out with Aeroqual portable indoor monitor device (a professional grade air quality measurement system) which had been pre-calibrated [36]. Three sensor nodes and the Aeroqual device were placed in a clear sealed glass container of 100 × 40 × 30 cm which was completely sealed. Then, the gas concentration inside the sealed container was made varied by injecting the particular gas of interest. The outputs of the sensors were recorded continuously for 1 h and plotted (Figure 8). Figure 8a shows that the result of NO_2_ sensor when 35 ppb of NO_2_ gas concentration was injected to the sealed container. It can be observed that the value for all sensor nodes including Aeroqual device gave relatively similar readings for 1 h period. During the experiment, the same room temperature setting at 25 °C was applied as shown in Figure 8b while Figure 8c shows the readings for humidity.

For validation purposes, means and standard deviations for all three parameters were calculated as shown in Table 3 below. Also shown, the mean value for NO_2_, temperature and humidity of three nodes (Node 1, Node 2 and Node 3) did not differ substantially between Aeroqual (a pre-calibrated device). This shows that the data measured for each developed sensor modules provide a similar response with the calibrated device. The standard deviation (Sd) from the table shows how the data differed from the mean value for each node. Overall, it shows that the developed system provides reliable data. 

**Table 3:** Means and standard deviations for gas and the sensors’ calibration.

Parameter	Node 1		Node 2		Node 3		Aeroqual	
	Mean	Sd	Mean	Sd	Mean	Sd	Mean	Sd
**NO_2_ (ppb)**	35.9	2.0	34.9	1.7	34.6	1.7	35.5	2.2
**Temperature (°C)**	25.6	0.3	26.5	0.1	25.7	0.1	25.7	0.1
**Humidity (%)**	39.7	0.8	39.6	0.6	39.3	0.6	40.2	0.1

### 6.2. Experimental Setup and Data Collection

**Figure 9:**
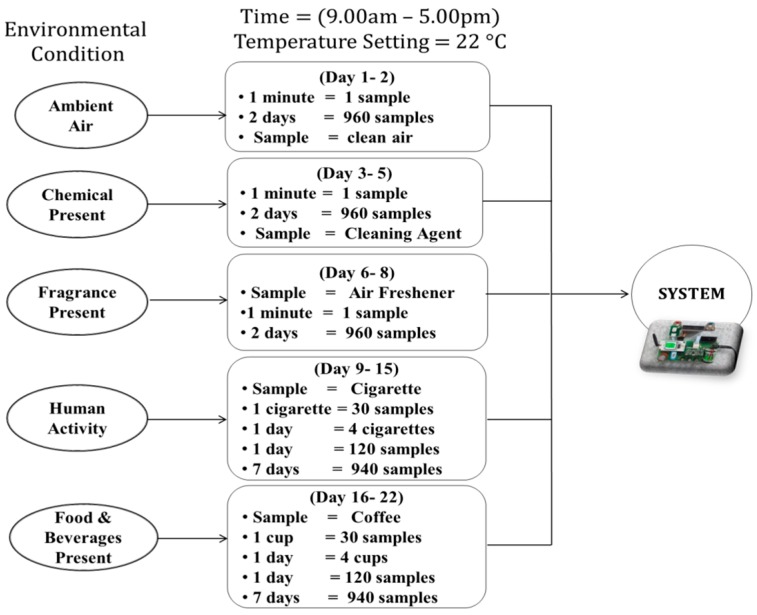
Data collection process.

The experiment was conducted in a medium-sized room of 4.5 m × 2.4 m × 2.6 m. There was an air-conditioner located at the center of the room at a height of 2.2 m from the ground. The sensor module was installed hanging up to the wall of the room at a height of 1.1 m from the ground. According to the Malaysian Standard on IAQ, the monitoring device or instrument should be positioned at a height between 75 cm and 120 cm, preferably 110 cm from the floor [37]. This position is considered as a breathing zone for the occupants. The node was powered up by using adaptor 7.5 V and was programmed to send the data to the base station every 1 minute. The data collection was conducted in 22 days between 9.00 a.m. and 5.00 p.m. with the room temperature set at 22 °C. Every day, after each experiment, the air in the room was purged out by opening windows to clean the air. Figure 9 shows the process of data collection for all five conditions from day 1–22.

The first condition was the ambient air environment. The purpose of this experiment was to collect the data of clean air for the room with the assumption that the ambient air was not contaminated. For the first environment, the data collection process took about two days. Thus, at the end of day 2, there were 960 samples collected for ambient air. The second environment was the environment with the presence of chemical substance. In this experiment, a cleaning agent was used as a proxy of the chemical substance. About 100 mL of chemical was put in a beaker and placed inside the room—at the centre of the room. The experiment was repeated for two days and 960 samples were collected during that period. For the third environment, an air freshener was used as a proxy to the fragrance. An automatic air freshener which released fragrance every 15 min was placed inside the room. It was hung up on the wall at a height of 2 m from the floor and about 2 m from the sensing node. The data was collected for two days with 960 samples. For the fourth condition of room environment with human activity, a person smoking a cigarette was chosen as a proxy. A person was asked to smoke in the room so that the real data of a person smoking cigarette in a room could be collected. That person smoked one cigarette at the centre of the room. Every cigarette produced data for approximately 30 min. The experiment was repeated four times a day for seven days. The amount of data collected for environment with human activity was 940 samples. Lastly, for the room environment with the presence of food and beverages, coffee had been selected to represent this category. A cup of coffee was placed in the middle of the room. Each cup of coffee manufactured 30 min of aroma. The experiment was repeated four times a day for seven days with 940 samples collected.

## 7. Result and Discussion

### 7.1. Sensor Response

**Figure 10:**
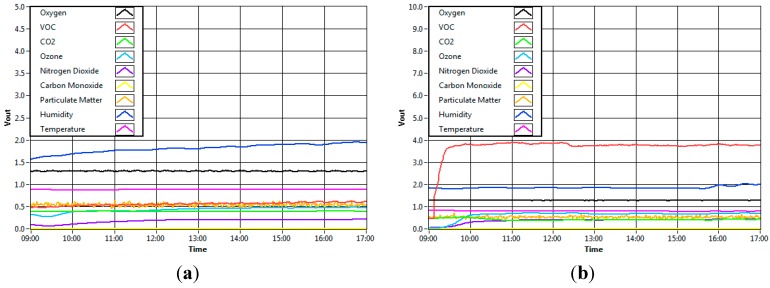

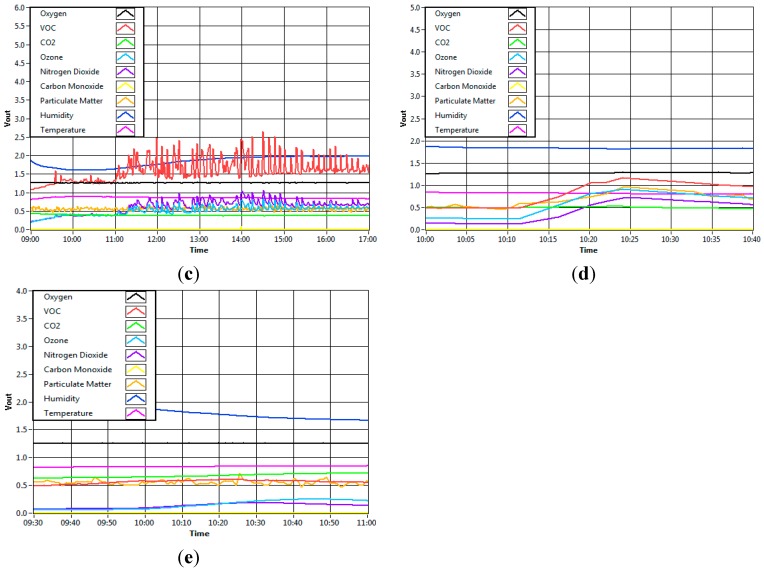
(**a**) Ambient environment; (**b**) Chemical presence; (**c**) Fragrance presence; (**d**) Human activity; (**e**) Food and beverages

Figure 10 below shows the sensors response toward five different environments which are ambient air, human activity, chemical presence, fragrance product (air freshener) and foods and beverages (coffee). The data was taken from one of samples of the experiments. They showed that the values of the sensors of the module give different responses to the environment. The response of the sensors indicated that the sensing module of the system functions accordingly to the varying sample concentration of different environment conditions. From the graphs, it is clear that the sensor node gave different reactions towards different environments. In Figure 10a, the sensors gave a relatively steady reading throughout the time. The sensors’ response was as expected since there was no substance which could interrupt the ambient air concentration. On the other hand, in Figure 10b, with the presence of chemical substance, it can be observed that certain gas sensors such as VOC, NO_2_ and O_3_ reacted differently as compared to ambient environment. The reading of VOC gas sensor particularly, rose sharply when the chemical was present in the room. Figure 10c represents the response of the sensors when the automatic air freshener released fragrance into the room every 15 min. The fragrance of the air freshener, however, vaporized quickly into the air after it was released. Thus, these changes of high and low concentration of fragrance in the air could be observed from the disturbed graph. Meanwhile, for the last two environments, 30 min of data were recorded instead of 8 h because these two environments had impact on the sensors for a short amount of time. Figure 10d illustrates the effect of the cigarette smoking activity on the sensor while Figure 10e denotes the presence of food and beverages (coffee) in the room. Notably, in all graphs, different sets of gas sensors reacted differently to different environments.

### 7.2. Principal Component Analysis (PCA)

**Figure 11:**
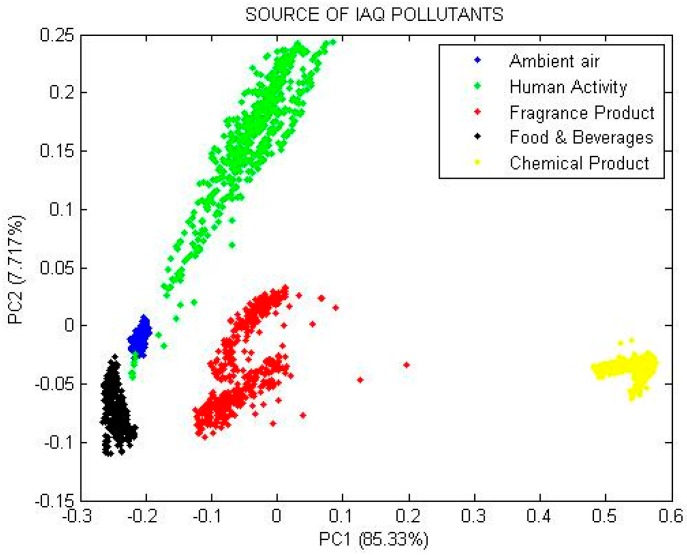
PCA plot of five different sources of IAQ pollutant.

The PCA was implemented to distinguish different varieties of the samples used in all environments. It is an unsupervised pattern recognition technique used to cluster the data according to groups. The techniques will reduce the size of data variable without losing the information [38]. Each principal component is a linear combination of the original variables as defined by the equation given below:

(2) $$PC_{p}=\;w_{1p}x_{1}+w_{2p}x_{2}+...+w_{np}x_{n}$$

where *PC_p_* is the notation for *p*-th order principal component for the overall *n* number of data, *W_np_* is regression coefficient (or weight) determined by PCA while *X_n_* is adjusted matric. The result of the PCA analysis is shown in Figure 11.

The scores of the five groups of sources of pollutants are plotted for principal component 2 (PC2) *versus* principal component 1 (PC1). Both components have two great discrimination of 85.330% and 7.717% (or total cumulative variance of 93.047%). From the PCA plot, the samples can be qualitatively clustered into five different groups based on different sources of pollutant. 

### 7.3. ANN Analysis

**Figure 12:**
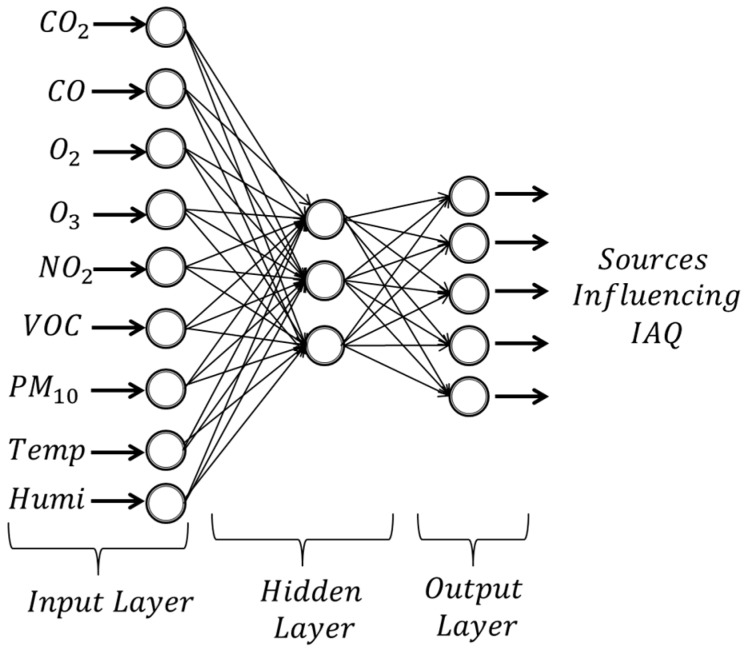
ANN model for source influencing IAQ.

In order to identify the source affecting the IAQ, this study used the multilayer feed-forward neural network which consists of three layers: input layer, hidden layer and output layer. As network architecture, a three-layer perceptron model as shown in Figure 12 was used. 

The first input layer contains the input variables for the network. Figure 12 illustrates that the input layer contains nine neurons of IAQ parameters which are CO_2_, CO, O_3_, NO_2_, O_2_, VOC, PM_10_, temperature and humidity. There is one hidden layer used and the number of hidden neurons is to be chosen in the model. The last layer of the model is the output layer which consists of five target outputs. After the system has been trained, it is expected that the system is able to identify the source influencing IAQ based on the five conditions. The ANN analysis was done using LabVIEW Machine Learning Toolkit (MLT) from National Instruments. The toolkit provides various machine learning algorithms in LabVIEW usually classified as supervised or unsupervised method. It is a powerful tool for pattern recognition, cluster identification and visualization of high-dimensional data. Some of classifiers function based on different ANN-algorithms such as multi-layer perceptron (MLP), self-organizing map (SOM), radial basis function (RB) and support vector machine (SVM) are available in this toolkit. For this research, the ANN-algorithm based on MLP with Back Propagation algorithm has been used to evaluate the system performance in the classification source influencing IAQ. The detailed parameter for ANN training is given in Table 4.

There are 4760 data collected and these data are gathered in a database as the training set. The database contains two data sets: 80% of data were used in training the network and 20% were designated as a testing set. The optimum structure of ANN model is determined by a trial and error method. The number of input neurons is 9, which corresponds to the number of sensors and the number of outputs is five neurons, which corresponds to the five environmental conditions. The number of hidden neurons was adjusted until the desired performance was achieved. In order to ensure convergence of the model, the input data was normalized within the range of [0, 1] based on Equation (3).

(3) $$X_{s^{\;}}=\;\frac{X-X_{min^{\;}}}{\left(X_{max}-\;X_{min^{\;}}\right)}$$

where *Xs* is the normalized value, *X*_min_ and *X*_max_ are the minimum and maximum value of input, respectively. The normalized data then was randomized and organized in as matrix input for training. An activation function was used to calculate the output response of a neuron. The sum of the input signal was applied with an activation to obtain the response. There are a number of common activation functions in use with neural networks. For this model, the hyperbolic tangent function was employed as the activation function. For each model, the network was trained 10 times. The mean, minimum and maximum classification rates are observed and recorded.

**Table 4:** Parameters for ANN training.

Training Parameter	Value
Sample Number of samples used for training: 3808 Number of samples used for testing: 952	4760
Input	9
Hidden neurons	Flexible
Output neurons	5
Performance	MSE
Goal	0.0001
Learning rate	0.01
Momentum constant	0.5

The results for the network model are tabulated and shown in Table 5. From Table 5, it can be observed that the model with network structure 9-15-5 gives the best accuracy with minimum and maximum classification accuracies of 98.8% and 100%, respectively. The result showed that the test classifications highly correlate with the sample data. The ANN model was able to classify the source that influenced IAQ and the classification rate was 99.1%.

**Table 5:** Results for network model.

Model Number	Model Structure	Mean Classification Accuracy		
		Minimum Classification (%)	Maximum Classification (%)	Mean Classification (%)
1	9-3-5	29.4	55.0	45.0
2	9-6-5	52.6	65.6	57.7
3	9-9-5	70.0	81.0	75.3
4	9-12-5	76.0	97.0	89.5
5	9-15-5	98.8	100.0	99.1

The confusion matrix for the model is shown in Table 6. Rows and columns represent actual and predicted values, respectively. While observing the confusion level of five different source of IAQ pollutant given in Table 6, it is observed that the chemical, fragrance, food and beverages presence has no confusion level. The human activity environment which is the presence of cigarette smoking has the highest confusion level compared to the other sources.

**Table 6:** Confusion matrix for five different sources of IAQ pollutant.

**Actual**	**Predicted**						
	**Sources of IAQ Pollutant**	**Ambient**	**Human Activity**	**Chemical**	**Fragrance**	**Food & Beverage**	**Accuracy (%)**
	Ambient	189	1	0	0	0	99.47
	Human Activity	6	184	0	0	0	96.84
	Chemical	0	0	191	0	0	100.00
	Fragrance	0	0	0	191	0	100.00
	Food & Beverages	0	0	0	0	190	100.00

## 8. Conclusions 

In this paper, an indoor air quality monitoring system has been developed with the additional function of classifying sources influencing the IAQ based on five different environments such as ambient air, chemical presence, fragrance presence, foods and beverages, and human activity. The data collection has been completed in 26 days in different environment simulations to obtain the desired effects of the five environments. The data for the ANN classification training was collected using a self-developed IAQ monitoring system. Each sensor module in the system contains nine sensors of gases and other parameters that are usually used in IAQ measurement like Carbon Dioxide, Carbon Monoxide, Ozone, Nitrogen Dioxide, Oxygen, Volatile Organic Compound and Particulate Matter, Temperature and Humidity. Based on the results for the network models, the ANN model modelled with network structure 9-15-5 was able to classify the sources influencing the IAQ with a minimum and maximum of 98.8% and 100%, respectively. On average, the system was about 99.1% correct. Overall, it can be concluded that the system delivered a high classification rate based on ANN.
